# Detection and Recognition of Asynchronous Auditory/Visual Speech: Effects of Age, Hearing Loss, and Talker Accent

**DOI:** 10.3389/fpsyg.2021.772867

**Published:** 2022-01-28

**Authors:** Sandra Gordon-Salant, Maya S. Schwartz, Kelsey A. Oppler, Grace H. Yeni-Komshian

**Affiliations:** Department of Hearing and Speech Sciences, University of Maryland, College Park, MD, United States

**Keywords:** auditory-visual speech perception, aging, hearing loss, foreign-accented speech, detection of asynchronous auditory-visual speech, recognition of asynchronous auditory-visual speech

## Abstract

This investigation examined age-related differences in auditory-visual (AV) integration as reflected on perceptual judgments of temporally misaligned AV English sentences spoken by native English and native Spanish talkers. In the detection task, it was expected that slowed auditory temporal processing of older participants, relative to younger participants, would be manifest as a shift in the range over which participants would judge asynchronous stimuli as synchronous (referred to as the “AV simultaneity window”). The older participants were also expected to exhibit greater declines in speech recognition for asynchronous AV stimuli than younger participants. Talker accent was hypothesized to influence listener performance, with older listeners exhibiting a greater narrowing of the AV simultaneity window and much poorer recognition of asynchronous AV foreign-accented speech compared to younger listeners. Participant groups included younger and older participants with normal hearing and older participants with hearing loss. Stimuli were video recordings of sentences produced by native English and native Spanish talkers. The video recordings were altered in 50 ms steps by delaying either the audio or video onset. Participants performed a detection task in which they judged whether the sentences were synchronous or asynchronous, and performed a recognition task for multiple synchronous and asynchronous conditions. Both the detection and recognition tasks were conducted at the individualized signal-to-noise ratio (SNR) corresponding to approximately 70% correct speech recognition performance for synchronous AV sentences. Older listeners with and without hearing loss generally showed wider AV simultaneity windows than younger listeners, possibly reflecting slowed auditory temporal processing in auditory lead conditions and reduced sensitivity to asynchrony in auditory lag conditions. However, older and younger listeners were affected similarly by misalignment of auditory and visual signal onsets on the speech recognition task. This suggests that older listeners are negatively impacted by temporal misalignments for speech recognition, even when they do not notice that the stimuli are asynchronous. Overall, the findings show that when listener performance is equated for simultaneous AV speech signals, age effects are apparent in detection judgments but not in recognition of asynchronous speech.

## Introduction

**FIGURE 1:**
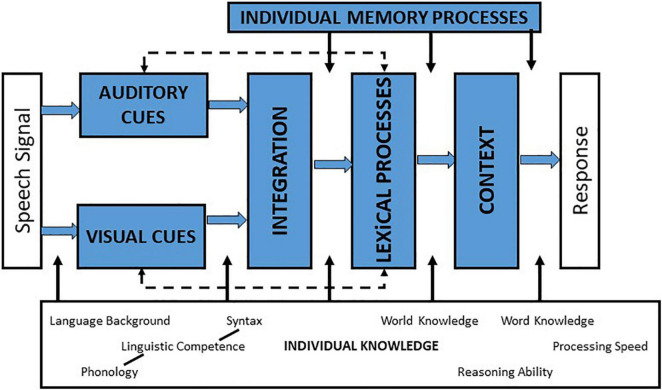
Schematic of a simple model of auditory-visual speech processing, adapted from Grant and Bernstein (2019) (Reproduced with permission from Springer publishers via the Copyright Clearance Center).

Everyday speech recognition tasks stimulate both audition and vision. Successful processing in both modalities requires accurate detection and resolution of auditory and visual cues at an early stage of processing, and binding of these separate streams of processed auditory and visual stimuli into a unified percept at one or more later stages of integration.] See AV integration model of Grant and Bernstein (2019), shown in Figure 1]. Auditory and visual features of speech stimuli are complementary to each other, and also provide some redundancy, both of which enhance a listener’s understanding of the speech signal and underscore the importance of accurate integration. Additionally, auditory-visual (AV) integration for speech signals is aided at multiple stages of processing by the listener’s knowledge of the language, as well as by the availability of contextual cues. Finally, the listener’s cognitive abilities contribute to the process of AV integration for speech. Specifically, working memory aids prediction about the spoken message as it unfolds over time, attention enables the listener to focus on the target message and ignore irrelevant information, and processing speed assists the listener in rapidly integrating, recognizing, and responding to a spoken message. [The reader is referred to Peelle and Sommers (2015), which proposes a dynamic process of AV integration consisting of early and later integration mechanisms in auditory cortex and posterior superior temporal sulcus, based on neurophysiological evidence].

One critical property for efficient integration of multisensory information is the temporal coherence between auditory and visual stimuli, which occurs naturally when these signals derive from the same source and have the same onset (Brooks et al., 2015). For naturally occurring speech signals, the relative onset of auditory and visual signals may not be perfectly aligned in time when it is received by the listener. For example, due to differences in the transmission speed of sound and light, the auditory signal arrives later than the visual signal when the talker is more than 10 m away from the receiver (Navarra et al., 2009). Visible speech information also arrives sooner than auditory information because preparatory movements of the jaw often precede speech production (Chandrasekaran et al., 2009; Schwartz and Savariaux, 2014). However, video signals transmitted through high-fidelity transmission (e.g., video presentation via television, streaming to a monitor or real-time remote face-to-face communication), may be prone to a lag in optical cues relative to acoustic cues (e.g., Grant et al., 2004). These examples of auditory-visual asynchrony are tolerated well by young listeners with normal hearing, who detect a range of asynchronies in auditory and visual speech signals as synchronous. Specifically, young normal-hearing listeners are relatively insensitive to asynchronies between about −50 ms (auditory lead/visual lag) to +150 ms (auditory lag/visual lead), such that there is a temporal window of approximately 200 ms over which asynchronous AV stimuli are detected as simultaneous. This window is referred to as the “AV simultaneity window” (Richards et al., 2017). The AV simultaneity window is remarkably robust, and has been observed for isolated nonsense syllables as well as for sentence-length materials (Grant et al., 2004). Additionally, the range of AV asynchronies over which young, normal-hearing adults maintain the same level of speech recognition performance, referred to as the “AV speech integration window,” is comparable to the 200 ms-wide AV simultaneity window, as measured with detection judgments (Grant and Seitz, 1998; Grant et al., 2004).

Advanced age may affect the efficiency of AV integration, particularly for asynchronous AV signals, because of age-related changes in auditory temporal processing. Older listeners exhibit slowed auditory temporal processing on simple measures of temporal acuity and duration discrimination (Fitzgibbons and Gordon-Salant, 1994; Snell, 1997), more complex tasks of duration discrimination in tonal sequences (Fitzgibbons and Gordon-Salant, 1994, 2001), and recognition of time-compressed speech (Gordon-Salant and Fitzgibbons, 1993). In contrast, advanced age does not appear to have a consistent effect on processing rate for visual information, with some studies reporting age-related delays on visual gap detection and temporal order judgment tasks (Humes et al., 2009; Busey et al., 2010) and others reporting a minimal effect of age on temporal processing of visual signals, depending on signal and task complexity (Brooks et al., 2015; Guest et al., 2015). Given that older listeners consistently show slowed auditory temporal processing but may not experience slowed visual processing, it might be expected that the auditory signal arrives later than the visual signal at the central integrator, resulting in a shift in the AV simultaneity window, possibly in the negative direction, during the AV synchrony/asynchrony detection task. To illustrate with a hypothetical example, an AV stimulus presented at −100 ms AV asynchrony indicates that the auditory signal is presented 100 ms before the visual signal (i.e., auditory lead) and may be perceived as out of sync by younger listeners. However, if there is slowed processing of that auditory signal by an older listener, then it may be perceived as synchronous with the visual stimulus; the simultaneous judgment at −100 ms would be seen as a shift in the AV simultaneity window in the negative direction, relative to that observed for younger listeners. It is noted that individuals with hearing impairment, either young or old, do not show deficits in auditory temporal processing beyond those attributed to age (Fitzgibbons and Gordon-Salant, 1994), suggesting that individuals with hearing impairment should exhibit similar patterns of AV integration (AV simultaneity windows and AV speech integration) as individuals with normal hearing when they are matched in age.

The effects of age and/or hearing loss on the detection of AV asynchrony are somewhat mixed. Hay-McCutcheon et al. (2009) reported that older listeners with normal hearing or who used cochlear implants exhibited more negative thresholds of asynchrony in the auditory lead/visual lag conditions than middle-aged listeners, but no threshold differences in the auditory lag/visual lead conditions. In contrast, Başkent and Bazo (2011) reported comparable AV simultaneity windows by young listeners with normal hearing and older listeners with hearing loss. Neither of these previous studies compared performance on the AV asynchrony detection task between younger and older listeners who were matched for hearing sensitivity, nor between listeners with normal hearing and hearing loss who were matched in age. The present study seeks to overcome these limitations by evaluating the performance of three listener groups: young listeners with normal hearing, older listeners with normal hearing, and older listeners with hearing loss, in an effort to tease out possible effects due to age separately from those attributed to hearing loss.

The model of AV integration efficiency proposed by Grant and Bernstein (2019) incorporates cognitive abilities that influence AV speech recognition performance at multiple stages of the integration process. Because advanced age is characterized by declines in working memory (Park et al., 2002), processing speed (e.g., Salthouse, 2009; Lipnicki et al., 2017), and attentional control (Carlson et al., 1995; Milham et al., 2002), possible deterioration of AV integration by older adults may be associated with declines in cognitive abilities. For auditory-only signals, recognition of noisy, accented, or fast speech by older listeners correlates with cognitive abilities, including attention/inhibition (Janse, 2012), processing speed (Füllgrabe et al., 2015; Gordon-Salant et al., 2016), and working memory (Rönnberg et al., 2008, 2013; Gordon-Salant and Cole, 2016). For AV speech integration tasks, it may be predicted that older people will require more time to perform a higher-level task, such as recognizing misaligned auditory and visual stimuli. Thus, age-related decline in processing speed may result in poorer speech recognition performance by older than younger listeners in asynchronous conditions. It is therefore hypothesized that the AV speech integration window of older listeners will be narrower than that observed for younger listeners. This prediction is supported, in part, by previous findings that older listeners (both with and without hearing loss) demonstrated significant declines in speech recognition (relative to maximum performance) in most auditory lead/visual lag conditions, but younger listeners rarely showed a decrement in these conditions (Gordon-Salant et al., 2017). In that study, processing speed was identified as the principal cognitive factor contributing to the variance in AV speech recognition scores. Two limitations of this prior study were that the range of auditory lag/visual lead asynchronies was quite limited, and that all listeners were tested at the same fixed SNR, resulting in different levels of overall performance by the three listener groups. The current study addressed these limitations by (1) presenting a broad range of AV asynchronies from −450 ms to +450 ms; and (2) testing each listener at an individually adjusted SNR to yield 70.7% correct performance in the speech recognition task (synchronous condition).

Foreign-accented speech is ubiquitous in contemporary society and is often characterized by differences in timing information compared to native-English speech, including alterations in vowel and sentence duration (Guion et al., 2000; Gordon-Salant et al., 2010a), lexical and suprasegmental stress patterns (Flege and Bohn, 1989; Trofimovich and Baker, 2006; Zhang et al., 2008; Gordon-Salant et al., 2015), and onsets of voicing in fricatives and affricates (Gordon-Salant et al., 2010a). In addition to these auditory-based changes with foreign-accented English, visible speech information may also be altered as a result of differences in speech production (Summers et al., 2010). There are few studies of AV integration with foreign-accented speech. At least one study has reported a reduced benefit of visual cues for recognition of foreign-accented speech relative to native English speech by younger listeners (Yi et al., 2013). In the auditory-only mode, older listeners exhibit considerable difficulty recognizing foreign-accented speech, which appears to be associated with the temporal modifications in foreign-accented English coupled with older listeners’ deficits in auditory temporal processing (Gordon-Salant et al., 2010b,^2013^, ^2015^). Thus, it is possible that the integration of auditory and visual information by older listeners is more challenging when recognizing foreign-accented speech than native English speech in conditions with auditory or visual delays, because older listeners will be less able to take advantage of visual and auditory cues that are misaligned to aid in resolving this type of speech signal. In other words, recognition of foreign-accented speech may be quite low in auditory lead and auditory lag conditions; the net effect is predicted to be a narrower AV speech integration window for foreign-accented speech than for native English. Further, it may be expected that older listeners with and without hearing loss will recognize foreign-accented speech more poorly in asynchronous AV conditions than younger listeners. Older listeners with hearing loss are expected to exhibit even narrower AV speech integration windows than older normal-hearing listeners, given the excessive difficulties of these listeners in recognizing foreign-accented speech (Gordon-Salant et al., 2013).

The overall objective of this investigation was to examine the extent to which slowed auditory temporal processing associated with advanced age is a source of altered AV integration, as assessed on tasks of detection and recognition of asynchronous AV speech. The influences of talker accent, listener hearing sensitivity, and cognitive abilities were also examined. The main experimental questions were: (1) do age and hearing sensitivity affect detection of AV asynchrony across a broad range of asynchronies? (2) Do age and hearing sensitivity affect recognition of AV asynchronous speech across a broad range of asynchronies? (3) Is there an effect of talker native language on listeners’ detection and recognition of asynchronous speech? (4) Do cognitive abilities affect the speech integration window? It was predicted that older listeners with and without hearing loss would exhibit negative shifts in the AV simultaneity window (as measured on the detection task) and narrower AV speech integration windows (as measured on the speech recognition task) relative to younger listeners. It was also expected that foreign-accented speech would result in a narrowing of both the AV simultaneity window and the AV speech integration window, particularly by older listeners. Finally, it was expected that processing speed and working memory would be the most important cognitive domains associated with recognition of asynchronous AV signals, consistent with previous research (Gordon-Salant et al., 2017). The results are expected to shed light on the impact of age and hearing loss on the ability to perceive AV signals, particularly when they are misaligned in time and spoken with a foreign accent, as is now commonplace.

## Materials and Methods

### Participants

**FIGURE 2:**
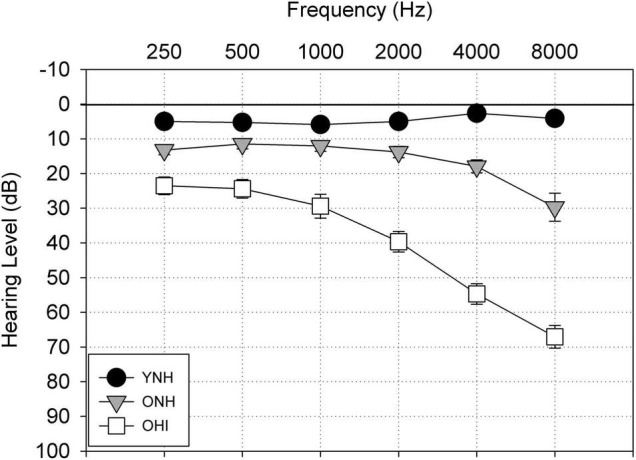
Mean audiometric thresholds from 250 to 8000 Hz in dB Hearing Level (re: ANSI 2018) for the young normal-hearing listeners (YNH), older normal-hearing listeners (ONH), and older hearing-impaired listeners (OHI). Error bars represent one standard error of the mean.

Listeners were recruited primarily on the basis of age and hearing sensitivity and were assigned to one of three groups of 17 listeners per group. A power calculation was conducted to determine the sample size with 80% power, significance level of 0.05, and an effect size (Cohen’s *d*) of 0.5, using mean and standard deviation data from a prior investigation of AV asynchrony (Gordon-Salant et al., 2017). The calculated sample size of 16 was increased by 1 to account for possible attrition. The young listeners with normal hearing (YNH; females = 11) were between 18 and 26 years of age (*Mean* = 20.8 years, *s.d*. = 50) and exhibited pure tone thresholds <25 dB HL (re: ANSI S3.6-2018, American National Standard Specification for Audiometers, 2018) between 250 and 4000 Hz. The older listeners with normal hearing (ONH; females = 15) fulfilled the same hearing criteria as the YNH listeners and were between 65 and 76 years of age (*Mean* = 70.1 years, *s.d*. = 0.87). The older listeners with hearing impairment (OHI; females = 3) were between 67 and 77 years (*Mean* = 72.0 years, *s.d. = 1.0*) and had a mild-to-moderate gradually sloping sensorineural hearing loss. The mean audiometric thresholds of the three listener groups are shown in Figure 2. Additional hearing criteria for all participants were monosyllabic word recognition scores of 80% or higher on Northwestern University Test No. 6 (Tillman and Carhart, 1966), normal tympanograms, and acoustic reflex thresholds present at levels consistent with data reported by Gelfand et al. (1990), indicative of normal hearing or a cochlear lesion (for the OHI listeners). Mean word recognition scores were 99.41, 99.29, and 94.6% for the YNH, ONH, and OHI listeners, respectively. All participants were native speakers of English and were required to pass a cognitive screening test (Montreal Cognitive Assessment, MoCA; Nasreddine et al., 2005) with a standard passing score of 26 or higher. They also were required to demonstrate normal visual acuity (20/40 or better), with or without correction.

### Stimuli

The stimuli were 720 IEEE sentences (Rothauser et al., 1969). Video recordings of all 720 sentences were made by three male native speakers of English (NE) and three male native speakers of Spanish (NS) at a professional recording studio (National Foreign Language Center, University of Maryland) using green-screen technology. Details of the recording procedures are reported in Waddington et al. (2020). Multiple speakers (rather than a single speaker) were used to increase the generalizability of the results. The speakers were all graduate students at the University of Maryland and ranged in age from 28–39 years. The native speakers of English had a general American dialect. The native speakers of Spanish came from South American countries (Peru, Argentina, and Chile) and moved to the United States after the age of 12 years. Ratings of their degree of accentedness by 10 YNH listeners indicated that they were all perceived as having a moderate Spanish accent (scores ranging from 4.95 to 5.90 on a scale of 1–9, with 1 indicating no accent and 9 indicating a heavy accent). The recordings were equated in root-mean-square (RMS) level across all speakers, and a calibration tone was created to be equivalent to this RMS level.

A six-talker babble consisting of spoken passages in English produced by three NE and three NS male talkers was used as the background noise. A description of the creation of this babble has been reported previously (Gordon-Salant et al., 2013). A calibration tone equivalent in RMS to the babble was also created.

Different lists of sentences were created for the three tasks administered in the experiment; these tasks are described in detail in the Procedures section: (a) the preliminary adaptive procedure, (b) the AV detection task, and (c) the AV recognition task. For the adaptive procedure, four sentence lists of 21 sentences each were created: two lists each of the NE talkers and two lists each of the NS talkers. These lists were used to determine the individual’s signal-to-noise ratio (SNR) prior to the detection task and prior to the recognition task.

For the detection task, there were three lists spoken by the three NE talkers (19 sentences/talker × 3 talkers = 57 sentences/list), and similarly, there were three lists spoken by the three NS talkers. Each of the 19 sentences spoken by each talker on a list was presented at a unique AV asynchrony, ranging from −450 ms (auditory lead) to +450 ms (auditory lag) in 50 ms steps (i.e., 19 asynchronies).

For the AV speech recognition task, 30 sentence lists were created with 15 NE lists and 15 NS lists. Each list consisted of four sentences spoken by each NE or NS talker, for a total of 12 sentences on a list, and featured a single AV asynchrony, ranging from −300 ms to +400 ms in 50 ms steps. None of the sentences were repeated between lists.

A custom-designed AV editing software application (Scenario Designer©, v. 1.5.3, created at the University of Maryland, College Park, MD, United States) was used to import the video files, scale and position the talker on the video monitor, and insert background babble. The media files for this application included the video recordings of the 720 sentences by each of the NE and NS talkers, as well as asynchronous versions of each of these videos. In the asynchronous version, the entire visual image (V) was manipulated to occur either before or after the onset of the audio signal. The talker was positioned in the center of the monitor and scaled for a full head and shoulders shot, with a solid blue screen inserted in the background. The six-talker babble was uploaded into the Scenario Designer software and used as the audio background noise.

### Procedures

Testing was conducted in a double-walled sound-attenuating booth at the University of Maryland. The Scenario Designer©software installed on a Mac computer controlled stimulus presentation and data collection. The speech and noise channels of the computer’s audio output were directed to separate channels of an audiometer (Interacoustics AC40, Eden Prairie, MN, United States). The levels of the speech and noise were controlled through the audiometer, with the speech level fixed at 85 dB SPL for all testing and the noise level varied individually, as described below. Calibration tones associated with the speech and noise were used to calibrate signal levels daily (Larson Davis 824 sound level meter with 2-cm^3^ coupler, Provo, UT, United States). Speech and noise signals were presented monaurally to the listener’s better ear through an Etymotic insert earphone (ER-3A). The video output of the Mac computer was displayed on a television monitor (32-inch Samsung television). The listener was seated 1-m from the television screen.

Three tasks were conducted multiple times over the course of the experiment: (1) the adaptive procedure; (2) AV asynchrony detection; and (3) recognition of AV asynchronous speech. In the adaptive procedure, synchronous AV sentences spoken by either the NE or NS talkers were presented in a background of 6-talker babble to the listener. The participants were asked to repeat the sentence. A two-down, one-up adaptive rule was applied, based on keyword accuracy (3 or more of 5 words correct → correct response), in which the babble level was adjusted to yield the SNR corresponding to 70.7% correct recognition (Levitt, 1971). The initial step size was 4 dB, which was reduced to 2 dB for sentences 5–21. The SNR corresponding to 70.7% correct recognition was determined following the procedures described for the Hearing in Noise test (HINT; Nilsson et al., 1994). The adaptive procedure was presented four times over the course of the experiment: once/each prior to the AV asynchrony detection task for the NE talkers and the NS talkers, and once/each prior to the AV recognition task with NE talkers and NS talkers. For each administration, lists developed for the adaptive procedure featuring the NE talkers were used prior to the detection and recognition tasks with NE talkers, and a comparable procedure was used for the tasks featuring the NS talkers. The adaptive procedure was repeated prior to the presentation of each experimental measure (detection or recognition) to ensure that the SNR was adjusted to yield 70.7% correct performance in the synchronous condition, immediately prior to the presentation of a new experimental task.

In the AV asynchrony detection task, lists of mixed synchronous and asynchronous AV sentences spoken by either the NE or NS talkers were presented in the babble adjusted to the SNR corresponding to the individual’s 70.7% correct recognition performance. After each sentence presentation, the listener was asked to respond “yes” if the auditory and visual presentation of the sentence was perceived as synchronous (in sync) and “no” if the auditory and visual presentation of the sentence was perceived as out of sync. The experimenter recorded each response. Each participant was presented with all AV asynchrony detection lists over the course of the experiment, resulting in nine judgments at each AV asynchrony for the NE talkers and nine judgments at each AV asynchrony for the NS talkers.

In the AV recognition task, 15 lists of sentences, each featuring a single AV asynchrony and spoken by either the NE or NS talkers (total of 30 conditions), were presented to listeners at the SNR corresponding to their 70.7% performance level for simultaneous AV signals. Recognition scores were derived as the percent of 60 keywords repeated correctly per list at each AV asynchrony. For each of the experimental tasks (detection and recognition), lists were blocked by talker accent and presented in randomized order across subjects.

Experimental testing was conducted over two visits, usually completed within 1 week. Each visit included the adaptive procedure, the detection task, a repeat of the adaptive procedure, and the recognition task. All tasks for the NE talkers were conducted during one visit, and all tasks for the NS talkers were conducted during the other visit, with the order of these visits randomized across subjects.

Listeners also were tested on a battery of cognitive measures. The cognitive measures assessed working memory [Listening SPAN (LSPAN), Daneman and Carpenter, 1980], processing speed [Digit Symbol Coding and Symbol Search from the Wechsler Adult Intelligence Scale (WAIS- III); Wechsler, 1997], inhibition (Flanker test from the NIH Toolbox, Eriksen and Eriksen, 1974), and executive function (Trail-making task, forms A and B, Reitan, 1958). The Flanker task and L-SPAN were administered via a tablet and PC, respectively, while all other cognitive measures were administered in a paper and pencil format.

The entire procedure was completed in approximately 4 h. Participants were compensated for their time in the experiment. This study involving human participants was reviewed and approved by the University of Maryland Institutional Review Board for Human Research. The participants provided their written informed consent to participate in this study.

## Results

### Signal-to-Noise Ratio Thresholds

**FIGURE 3:**
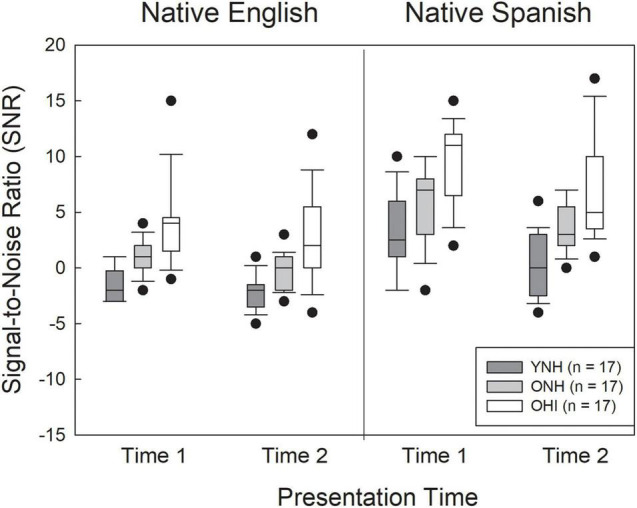
Box plots for signal-to-noise ratios (SNRs) corresponding to approximately 70.7% correct recognition for the young normal-hearing listeners (YNH), older normal-hearing listeners (ONH), and older hearing-impaired listeners (OHI) listeners for native English and native Spanish talkers. Medians: Inside box lines. Upper and lower quartiles: top and bottom edges of the box, respectively. The endpoints of the whiskers represent the range of values without the outliers.

Initial data analysis examined the SNR values corresponding to approximately 70% correct recognition, obtained prior to the detection and recognition tasks. Box plots showing the SNR results (medians, upper and lower quartiles, upper and lower extremes) for the three listener groups in the two test administrations for both the NE and NS talkers are shown in Figure 3. The figure shows that the three listener groups performed differently from each other, and the SNRs were lower (better) for the NE than the NS talkers. In addition, SNR values tended to decrease from the first test administration to the second. An analysis of variance (ANOVA) was conducted on listener SNR values in a split-plot factorial design with two within-subjects factors (test time, talker accent) and one between-subjects factor (group). Results revealed significant main effects of listener group [*F*(2,47) = 24.53, $\eta_{\text{p}}^{2}$ = 0.51, *p* < 0.001], talker accent [*F*(1,47) = 119.79, $\eta_{\text{p}}^{2}$ = 0.48, *p* < 0.001], and test time [*F*(1,47) = 43.50, $\eta_{\text{p}}^{2}$ = 0.72, *p* < 0.001]. There was also a significant accent by time interaction [*F*(1,47) = 5.41, $\eta_{\text{p}}^{2}$ = 0.10, *p* = 0.024]. None of the other interactions were significant. *Post hoc* analysis (Bonferroni) of the group effect showed that the YNH listeners had lower SNRs than the ONH listeners (*p* = 0.01) and the OHI listeners (*p* < 0.001), and that the ONH listeners had lower SNRs than the OHI listeners (*p* < 0.001). It is likely that the slight differences in hearing threshold between the YNH and ONH listeners, and the substantial threshold differences between the ONH and OHI listeners, accounted for this pattern of group effects. The source of the interaction between talker accent and test time appears to be a greater change in performance from test time one to test time two for the NS talkers (mean SNR difference = 2.36, *t* = 5.9, *Cohen’s d* = 0.83, *p* < 0.001) than for the NE talkers (mean difference in SNR = 1.26, *t* = 4.3, *Cohen’s d* = 0.61, *p* < 0.001). Listener performance for both NE and NS talkers improved (SNRs lower) in test administration two compared to test administration one.

### Auditory-Visual Asynchrony Detection

**FIGURE 4:**
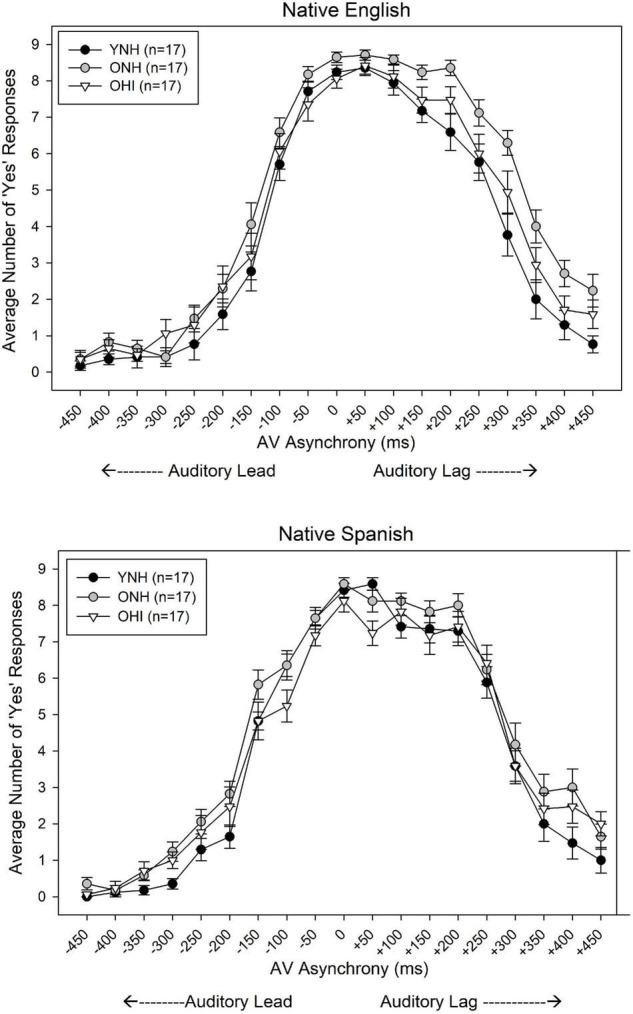
Mean ‘yes’ responses (total possible = 9) for each degree of AV asynchrony, ranging from –450 ms (auditory lead) to +450 ms (auditory lag), obtained from YNH, ONH, and OHI listeners for native English talkers **(Top)** and native Spanish talkers **(Bottom)**. Error bars represent one standard error of the mean.

The mean AV asynchrony detection judgments of the three listener groups are shown in Figure 4. The data are plotted as number of “yes” responses, indicating the AV stimulus was perceived as synchronous, out of a total of nine presentations for each AV asynchrony. As expected, listeners of all three groups generally perceived stimuli in the 0 ms AV condition as synchronous, for both unaccented (NE) and accented (NS) talkers. Additionally, the mean AV simultaneity windows are asymmetric around the synchronous (0 ms AV) condition, with listeners showing greater sensitivity (perceiving asynchronies) for auditory lead/visual lag stimuli compared to auditory lag/visual lead stimuli, as reported by others (Grant et al., 2004).

The approach to data analysis for both the detection and recognition judgments was guided by the goal of comparing data for each type of judgment to data reported previously. To facilitate these comparisons, analyses were selected that would enable determination of the lead and lag conditions in which performance was significantly different from the simultaneous (0 ms) condition. Subsequently, the AV simultaneity windows for detection and the AV speech integration windows for recognition could be determined. To that end, the AV asynchrony detection judgments were analyzed with a model building approach (Hox et al., 2017) using generalized linear mixed effects regression analysis (glmer) in the lme4 package with R studio software (Bates et al., 2015). The dependent variable was the binary response (synchronous, coded as 0, and asynchronous, coded as 1) for each trial of AV stimulus presentation. Initial full model testing included all fixed factors of talker accent (dichotomous variable, coded as 0 = NE talker and 1 = NS talker), group (categorical variable, coded as 0 = YNH, 1 = ONH, 2 = OHI), and AV asynchrony conditions [(19 AV conditions, ranging from −450 ms to +450 ms, each tested dichotomously with 0 = synchronous condition (0 ms), 1 = specific negative or positive asynchronous condition], as well as all interactions between these main effects. The random effects of participant and sentence, as well as random slopes of asynchrony by participant, also were included in the model. The full model that converged was referenced to YNH listeners, NE talkers, and the 0 ms (synchronous) AV stimulus presentation. The model included the random effect of participant and significant fixed effects of AV asynchrony between −450 and −50 ms, and between +150 and +450 ms (based on the Wald ratio *z*-statistic in the model output, which compares the coefficient’s estimated value with the standard error for the coefficient when data are normally distributed). The fixed effects of listener group and talker native language were not significant (*z* > 0.05). However, there were significant two-way interactions between talker native language and listener group at seven asynchronies (−450, −300, −250, −200, +300, +350, and +450 ms), and several three-way interactions between listener group, talker native language, and AV asynchrony. Results of the model output are shown in the Supplementary Material (interactions that were not significant removed to save space).

Because the variation in the AV simultaneity window for the two types of talkers for each listener group was of primary interest, the three-way interactions were explored further. To that end, subsequent general linear mixed effects analyses were conducted in which the reference listener group and talker’s native language were re-leveled. A significance level of *z* < 0.01 from the model output was applied to determine which AV asynchronies were detected as significantly different from simultaneity (0 ms). This strategy permitted an assessment of the range of AV asynchronies over which detection performance was not significantly different from maximal performance (at simultaneity) separately for each listener group and talker type. Table 1 shows the results of these analyses, including the minimum auditory lead/visual lag condition (most negative asynchrony) at which detection performance was not significantly different from simultaneity, the maximum auditory lag/visual lead condition (most positive asynchrony) at which performance also did not differ significantly from synchrony, and the difference between these two values (i.e., the AV simultaneity window). Three findings are apparent: (1) the AV simultaneity window of the YNH listeners did not differ for the NS and NE talkers; (2) the AV simultaneity window of the two older groups was narrower for the NS talkers than for the NE talkers; and (3) for the NE talker, the width of the AV simultaneity window was wider for the two older groups than for the younger group.

**TABLE 1:** Minimum auditory lead and maximum auditory lag asynchronies (in ms) at which detection of AV asynchrony of three listener groups was not significantly different from detection of simultaneous AV stimuli.

Group	Talker	Auditory Lead	Auditory Lag	AV Simult. Window
YNH	NE	0 ms	100 ms	100 ms
	NS	−50 ms	50 ms	100 ms
ONH	NE	−50 ms	200 ms	250 ms
	NS	0 ms	200 ms	200 ms
OHI	NE	−50 ms	200 ms	250 ms
	NS	0 ms	100 ms	100 ms

*Also shown is the AV simultaneity window, in ms. YNH, young normal hearing; ONH, older normal hearing; OHI, older hearing-impaired. NE, native English talker; NS, native Spanish taker.*

### Auditory-Visual Recognition

**FIGURE 5:**
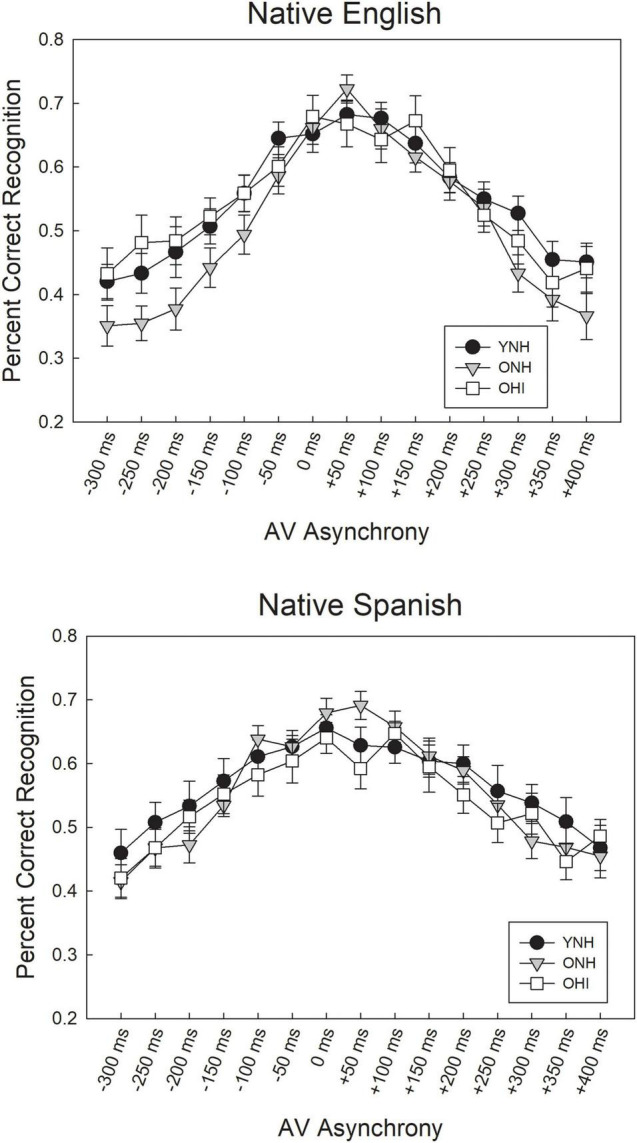
Mean speech recognition scores for asynchronous AV IEEE sentences, ranging from –300 ms (auditory lead) to +400 ms (auditory lag), obtained from YNH, ONH, and OHI listeners for native English talkers **(Top)** and native Spanish talkers **(Bottom)**. Error bars represent one standard error of the mean.

Recognition scores for the three listener groups in the 15 synchrony/asynchrony conditions for the NE and NS talkers are shown in Figure 5. The SNR adaptive procedure was successful in equating the three listener groups in the 0 ms AV (synchronous) condition at approximately 70% correct level of performance, as confirmed by one-way ANOVAs indicating no significant performance differences between the three groups for either the NE talkers [*F*(2,50) = 0.169, *p* = 0.845] or the NS talkers [*F*(2,50) = 0.773, *p* = 0.467]. Mean speech recognition scores in the synchronous condition were between 66 and 70% across groups and talkers, indicating a close approximation to the target 70.7% recognition score.

A statistical model was fit to the sentence recognition data using the glmer analysis, following the model building approach described above (Hox et al., 2017). The first iteration of model building included random effects of participant and sentence, and random slopes of asynchrony by participant (to examine variation in participants by level of AV asynchrony), as well as fixed effects of listener group, talker native language and degree of asynchrony and all interactions between these effects. The dependent variable was the trial-by-trial number of keywords correct out of five possible, for each sentence presented. Initial model testing included all fixed factors of interest: talker accent (dichotomous variable, coded as 0 = NE talker and 1 = NS talker), group (categorical variable, coded as 0 = YNH, 1 = ONH, 2 = OHI), and AV asynchrony conditions [15 AV conditions, ranging from −300 ms to +400 ms; each tested dichotomously with 0 = synchronous condition (0 ms), 1 = specific negative or positive asynchronous condition], as well as their interactions. The referent talker accent was NE, referent group was YNH, and referent AV asynchrony condition was 0 ms. Model testing proceeded with iteratively removing the highest-order fixed effects and interactions that were not significant (*z*-statistic > 0.05 in the model output), and re-running the model. Improvement in model fit between the full model and subsequent models was assessed with the ANOVA test.

The best-fitting model derived from these fixed and random effects, shown in Table 2, included the random effects of participant and sentence item, and the fixed effect of asynchrony condition. The fixed effects of listener group and talker native language and all interactions were not significant and subsequently were removed from the final model. With reference to the 0 ms (synchronous) condition, each fixed asynchrony condition was significantly different (*z* < 0.001), with the exception of the +50 and +100 ms AV asynchronies, as shown in the table. That is, performance in each negative AV asynchrony condition (−50 ms through −300 ms) was significantly different from the synchronous condition (0 ms), and performance for the positive AV asynchronies between +150 ms through +400 ms was also significantly different from the synchronous condition. Overall, the results suggest that the AV speech integration window for sentence recognition, based on the range between the minimum negative and minimum positive asynchronies where performance was not significantly different from 0 ms, was between 0 ms to +100 ms, or 100 ms wide, and was similar for all three listener groups and for both NE and NS talkers.

**TABLE 2:** Final model of YNH, ONH, and OHI listener speech recognition performance.

	Coefficient	*SE*	*z*	*p*
Intercept	0.84	0.17	5.06	<0.001
AV asynchrony − 300	−1.71	0.21	−8.03	<0.001
AV asynchrony − 250	−1.51	0.21	−7.20	<0.001
AV asynchrony − 200	−1.44	0.21	−6.91	<0.001
AV asynchrony − 150	−1.17	0.21	−5.68	<0.001
AV asynchrony − 100	−0.99	0.20	−4.82	<0.001
AV asynchrony − 50	−0.46	0.21	−2.23	<0.001
AV asynchrony + 50	0.41	0.22	1.84	>0.05
AV asynchrony + 100	−0.12	0.21	−0.55	>0.05
AV asynchrony + 150	−0.42	0.21	−2.03	<0.05
AV asynchrony + 200	−0.42	0.21	−2.03	<0.05
AV asynchrony + 250	−0.66	0.20	−3.24	<0.01
AV asynchrony + 300	−1.23	0.21	−5.96	<0.001
AV asynchrony + 350	−1.40	0.21	−6.71	<0.001
AV asynchrony + 400	−1.51	0.21	−7.19	<0.001
Talker NS × Asynch − 100	0.84	0.29	2.86	<0.01
Talker NS × Asynch − 100 × YNH	−0.89	0.42	−2.13	<0.05

### Predictors of Recognition Performance

Mean scores (and standard errors) on the six cognitive measures for the three listener groups are shown in Table 3. ANOVAs were conducted separately for each of these measures and revealed a significant effect of listener group on each test [Digit Symbol: *F*(2) = 37.05, $\eta_{\text{p}}^{2}$ = 0.61, *p* < 0.001; Symbol Search: *F*(2) = 22.13, $\eta_{\text{p}}^{2}$ = 0.48, *p* < 0.001; LSPAN: *F*(2) = 14.95, $\eta_{\text{p}}^{2}$ = 0.384; Trail Making A: *F*(2) = 10.97, $\eta_{\text{p}}^{2}$ = 0.314, *p* < 0.001; Trail Making B: *F*(2) = 11.78, $\eta_{\text{p}}^{2}$ = 0.329, *p* < 0.001; Flanker (uncorrected): *F*(2) = 28.59, $\eta_{\text{p}}^{2}$ = 0.55, *p* < 0.001]. *Post hoc* multiple comparison tests using the Bonferroni correction revealed that the YNH listeners had significantly higher scores than the two older listener groups on the Digit Symbol, Symbol Search, LSPAN, and Flanker tests, and significantly lower scores than the two older groups on the Trail Making A and B tests. However, there were no differences in the performance between the two older groups on any measure (*p* > 0.05, each measure).

**TABLE 3:** Mean scores (and standard deviations) of the three listener groups on the six cognitive measures.

	YNH		ONH		OHI	
	Mean	*s.d.*	Mean	*s.d.*	Mean	*s.d.*
Digit symbol	90.11	11.90	62.48	13.10	55.35	12.31
Symbol search	41.94	7.09	28.29	6.91	28.23	6.04
LSPAN	4.21	1.25	2.79	0.81	2.68	0.50
Trail making A	18.77	5.02	26.32	7.54	29.24	7.33
Trail making B	35.90	9.60	60.01	19.49	71.92	31.39
Flanker	112.81	5.18	97.35	7.91	98.59	6.01

The best-fitting model for the asynchronous AV sentence recognition scores, described above, was next probed to determine which predictor variables of cognition and hearing sensitivity improved the model fit (based on the ANOVA test). Model testing proceeded from the reduced model described above to subsequently include, in separate iterations, each of the predictor variables (all continuous variables): working memory (L-SPAN), speed of processing (Digit Symbol Coding, Symbol Search), attention/inhibition (Flanker score), executive function (Trail Making A and B), pure-tone hearing thresholds [quantified as pure-tone average of thresholds at 0.5, 1, and 2 kHz (the PTA), and as high-frequency pure-tone average of thresholds at 1 k, 2 k, and 4 kHz (the HF-PTA)]. Scores were converted to *z*-scores prior to entering each variable sequentially into the model. None of the predictor variables improved model fit.

Finally, an analysis was conducted to determine the cognitive and hearing sensitivity variables that best predicted the SNRs at which listeners achieved 70% correct performance for NE and NS speech in the simultaneous AV condition. Because significant differences in these SNR values were observed between the first and second administrations, separate analyses were conducted for each of the four dependent measures (NE and NS speech, time 1 and time 2). Linear multiple regression analyses were conducted using a reduced set of predictor variables to minimize the effects of multicollinearity. The predictor variables were the Digit Symbol Coding, LSPAN, Flanker-uncorrected, and Trail Making A tests, and HF-PTA. Results of the linear regression analyses with the step-wise method are shown in Table 4, and revealed that for each SNR measure, the predictor variable that accounted for the most variance was HF-PTA. The Trail Making A test of executive function accounted for additional variance in recognition of NE speech in the first administration, and the LSPAN test of working memory accounted for additional variance for recognition of NS speech in both the first and second test administrations.

**TABLE 4:** Results of linear multiple regression analyses with three predictor variables retrieved (HF-PTA, Trail Making A, and LSPAN).

Variables retrieved	SNR condition			
	NE speech		NS speech	
	Time 1	Time 2	Time 1	Time 2
HF-PTA (1)	0.559	0.546	0.697	0.757
Trail making A (2)	0.635	–		
LSPAN (2)			0.741	0.791

*Cumulative variance (r^2^) accounted for by significant predictor variables, in the order retrieved by stepwise multiple linear regression [first (1), second (2)], is shown for SNRs measured at two test intervals for native English (NE) and native Spanish (NS) talkers. Criteria for significance of each retrieved variable in the table is p < 0.05, and the significance of each regression model associated with each retrieved variable is p < 0.001.*

## Discussion

This study evaluated whether older listeners with and without hearing loss exhibit different patterns of AV integration for asynchronous auditory-visual sentences compared to younger listeners with normal hearing, and whether such patterns were influenced by talker accent and task. Results generally showed that detection of AV asynchrony varied between younger and older listeners, with different patterns observed for talkers of different native language backgrounds. However, for the speech recognition task, all three listener groups showed comparable effects of AV asynchrony for both NE and NS talkers. These findings and their interpretation are explained in more detail below.

### Auditory-Visual Asynchrony Detection

Younger and older listeners showed different AV simultaneity windows across the range of AV asynchronies assessed, and these patterns varied with talker native language. For the native English talker, the minimum auditory lead condition perceived as synchronous was more negative for the ONH and OHI listeners compared to the YNH listeners. The negative shift, on average, was 50 ms for both the ONH and OHI listeners, consistent with the hypothesis that slowed auditory temporal processing, but not visual processing, by older listeners may have delayed perception of the auditory stimulus. That is, an auditory stimulus presented prior to a visual stimulus was perceived as more closely aligned in time to the visual stimulus by older listeners than younger listeners, imposing an overall negative shift in the AV simultaneity window. The findings also showed that the derived AV simultaneity window for NE talkers was at least twice as wide for older listeners than younger listeners. It appears that older listeners were less sensitive to AV asynchronies in the auditory lag/visual lead conditions than the younger listeners, which contributed to the differences in the width of the AV simultaneity window. The negative shift in the asynchronous AV stimuli in auditory lead conditions that are perceived as simultaneous by older listeners is consistent with findings reported by Hay-McCutcheon et al. (2009). While both the current study and that of Hay-McCutcheon et al. (2009) showed a wider range of AV asynchronies as judged as simultaneous for older listeners than younger listeners, the source of the increased width was different in the two studies. Hay-McCutcheon et al. (2009) attributed the increased range to more negative auditory lead thresholds exclusively, whereas the source of the wider windows of older listeners in the current study is associated with both more negative auditory lead asynchronies and more positive auditory lag/visual lead asynchronies yielding comparable detection performance to that observed for simultaneous AV stimuli. The increase in the positive side of the AV simultaneity window among older listeners compared to younger listeners may reflect less sensitivity to visually leading AV stimuli. This decreased sensitivity for visual leading asynchronous AV speech may serve older listeners well for situations where there is a delay in electronic transmission of auditory signals relative to visual signals, such as with hearing aids or with internet communication. The present results, however, are in contrast to findings of Başkent and Bazo (2011), who reported no differences in the AV simultaneity window between younger listeners with normal hearing and older listeners with hearing loss. One possible source of this discrepancy may be the method used to determine the range of AV asynchronies that are judged to be comparable to maximum performance (observed for simultaneous AV stimuli). Başkent and Bazo (2011) measured the AV simultaneity window encompassed by the 50% threshold points for auditory-leading and visual-leading stimuli, whereas the current study measured the window encompassed by AV asynchronies that were judged as not significantly different from simultaneous AV stimuli. Thus, different methods for calculating the AV simultaneity window may yield discrepant findings; the current method was chosen to facilitate comparison between detection and recognition judgments.

The AV simultaneity windows were narrower for sentences spoken by NS talkers compared to those spoken by NE talkers for the older listeners, but not for the younger listeners. This difference was attributed to a change in the asynchrony detection threshold for auditory leading signals for both older listener groups, which shifted in a more positive direction for NS talkers relative to NE talkers, suggesting that older listeners became more sensitive (i.e., perceived asynchrony) in auditory lead conditions for the more challenging NS talkers. Additionally, the OHI listeners’ judgments for visual leading signals (positive AV asynchronies) shifted in a more negative direction, indicating that these listeners also were highly sensitive to asynchronies for NS talkers in visual lead conditions. While the source of the narrower AV simultaneity windows by the older listeners is not known, one possible explanation is that these listeners were so challenged by the Spanish-accented speech that they paid more attention to the visual stimuli to recognize the sentence, and as a result, disparities in the relative onset of auditory and visual information became more obvious. It should be noted that the AV simultaneity window for the native English talkers was somewhat narrower than the typical 200 ms integration window reported in other studies for younger listeners (e.g., Grant et al., 2004). Differences in method across the different studies likely accounted for the variation in window size, including the use of an adaptive procedure to equate listener performance prior to measuring the detection thresholds for asynchronous stimuli in the current study, as well as the use of multiple NE and NS talkers and a babble background.

### Auditory-Visual Recognition

Recognition of synchronous and asynchronous AV sentences was examined to determine whether or not listeners’ recognition performance is affected by asynchronous presentation of AV stimuli, and whether possible differences in AV integration between younger and older listeners impact performance on this task The results generally show that all listener groups exhibited significant declines in recognition performance for asynchronous presentation of AV sentences, when recognition performance was equated for synchronous speech. However, contrary to expectation, statistical modeling of the sentence recognition scores failed to reveal effects of listener age or hearing loss. It was expected that older listeners would show significantly greater declines in performance in the auditory lead conditions compared to younger listeners, as was shown in a previous study (Gordon-Salant et al., 2017). In auditory lead conditions, the lips are clearly misaligned with the talker’s voice, requiring listeners to inhibit the distracting effect of poor bi-sensory signal alignment. It was expected that older listeners, who often have a compromised ability to inhibit irrelevant or distracting stimuli (e.g., Hasher and Zacks, 1988; Alain and Woods, 1999; Presacco et al., 2016) would be more impacted by such temporal onset misalignments that are perpetuated through the duration of the sentence. In a previous experiment (Gordon-Salant et al., 2017), the younger listeners performed near ceiling for the synchronous sentence stimuli and maintained a high level of performance for all asynchronies, indicating that they were minimally impacted by the temporal misalignments. However, the ONH and OHI listeners’ recognition performance for synchronous AV speech was considerably poorer than that of the YNH listeners, and these two older groups showed significant declines in recognition in auditory lead conditions. It appears, then, that the current technique of equating listener performance to the same level in the 0 ms AV synchronous condition is critically important for evaluating the extent to which age and hearing loss, *per sé*, affect the AV speech integration window. This is reinforced by the observation that performance of YNH, ONH, and OHI listeners at the same fixed SNR will be inherently different, with one group or another performing at or near the ceiling or floor, making it difficult to observe the differential impact of the asynchronous distortions on recognition performance by the different listener groups. The finding that older listeners did not exhibit significantly greater declines in speech recognition than younger listeners in auditory lead conditions in the current study may also reflect the effects of slowed auditory processing among older listeners. That is, if processing of the auditory information in auditory lead conditions is delayed among older listeners, then the auditory signal may appear more synchronous with the visual signal, and relatively high recognition performance is maintained. Further investigation of this possible mechanism is warranted, using more discrete steps of the asynchronous stimulus presentation.

The statistical model of asynchronous AV sentence recognition performance also failed to show an effect of talker native language. Thus, listeners showed the same pattern of decline in speech recognition scores with AV asynchronies for both the NE and NS talkers. This finding was also contrary to expectation, as recognition of the asynchronous sentences spoken by NS talkers was expected to be extremely challenging, especially for older listeners. The method of equating performance for simultaneous AV stimuli separately for the NS and NE talkers likely reduced the expected performance declines for asynchronous NS sentences. The final model revealed that for both talker accents, recognition performance in the auditory lead conditions between −50 and −300 ms was significantly different from performance in the synchronous condition. Similarly, recognition performance in the visual lead/auditory lag conditions between +150 and +400 ms was significantly different from the synchronous condition. Based on these results, the AV asynchronies over which listener performance was comparable to that observed in the simultaneous AV condition (i.e., the AV speech integration window) was 100 ms wide, for speech produced by both NE and NS talkers. This window is comparable to the AV simultaneity window identified in the AV detection task for NE and NS talkers for young normal-hearing listeners. However, for older listeners, the AV integration window observed for recognition judgments was narrower than the AV simultaneity window on the detection task, particularly for the NE talker. Taken together, these results suggest that even though older listeners may be relatively insensitive in detecting asynchronies in AV speech stimuli, the same stimuli have a deleterious impact on recognition performance. In contrast, younger listeners appear to have difficulty accurately recognizing asynchronous AV sentences at the same asynchronies where they detect the presence of asynchrony.

These findings have implications for everyday communication. For example, there are many face-to-face interactions in daily life where the auditory and visual speech information may be misaligned in time, including internet communication (i.e., Zoom meetings), television programming, excessive distance between talker and receiver, or electronic amplification of the talker’s speech with additional signal processing (see Gordon-Salant et al., 2017 for a review). The current findings suggest that all listeners, regardless of age and hearing loss, may have considerable difficulty accurately recognizing such asynchronous signals. Although the older listeners did not exhibit significantly poorer recognition performance or different speech integration windows than the younger listeners, these findings may not reflect age-related performance patterns in everyday listening situations, where the SNR is not individually adapted, but rather is similar for all listeners, depending on their location in the auditory scene.

Cognitive measures and hearing sensitivity were not significant predictor variables for recognition of asynchronous AV sentences. Two possibilities may account for these findings. The first is that the specific measures used to quantify cognitive ability were not sufficiently sensitive to identify individual variation. The second is that there was not sufficient variation in speech recognition performance among the listeners in the asynchronous AV conditions, because listener performance was equated in the synchronous condition. A related issue is the method of setting SNR prior to testing perception of asynchronous speech. A previous study of age-related differences in recognition of asynchronous AV stimuli (Gordon-Salant et al., 2017) presented asynchronous AV stimuli at the same SNR to all listeners, with some groups performing near ceiling and other groups performing near floor for particular stimuli. In that study, the cognitive measure of speed of cognitive processing contributed to variation in recognition performance in asynchronous conditions. Thus, the results are very different when stimuli are presented at the same fixed SNR to all participants vs. when stimuli are presented at an individually adjusted SNR to equate performance level across participants. Comparing the present findings with those reported previously (Gordon-Salant et al., 2017) tentatively suggests that the method of setting SNR, and the resulting level of recognition performance for synchronous AV signals, are important factors in determining the impact of cognitive abilities on recognition of asynchronous AV speech signals presented in noise. That is, adjusting the SNR on an individual basis may have provided compensation for the effects of cognitive decline or hearing sensitivity, or both. It appears that these predictors may be relevant when the SNR is fixed, as in many everyday listening situations.

### Signal-to-Noise Ratio Thresholds

The SNRs corresponding to 70.7% correct recognition performance were significantly better during the second administration compared to the first administration for both NE and NS talkers. The adaptive measure was conducted twice with the same talkers (NE or NS) on each test day in order to equate performance immediately prior to the administration of the detection task and the recognition task. The improvement in SNR threshold in the second administration on the same test day may reflect, in part, a simple effect of learning the task (as sentences were not repeated). However, performance improved significantly more for the NS talkers than the NE talkers (effect size was strong for NS talkers and moderate for NE talkers), and may be one manifestation of rapid adaptation to foreign-accented speech reported previously (Clarke and Garrett, 2004; Bradlow and Bent, 2008; Gordon-Salant et al., 2010c; Bieber and Gordon-Salant, 2021). The effect of listener group did not interact with test time nor talker accent, indicating that both groups showed the same improvement with the second administration of the adaptive procedure. The rapid improvement in recognition of foreign-accented speech by younger and older listeners is consistent with previous findings (Gordon-Salant et al., 2010c; Bieber and Gordon-Salant, 2017) and is underscored here as a variable to control in future studies that employ multiple presentations of foreign-accented speech. Listener high-frequency pure-tone average accounted for the most variance in SNR scores across the two test administrations and for NE and NS speech. These results are highly consistent with prior findings of the importance of hearing sensitivity for recognition of speech in noise (e.g., Humes and Dubno, 2010), as well as the importance of working memory for recognition of degraded speech (Rönnberg et al., 2013), when the speech signals are presented in the auditory mode. However, the current findings extend these principles to recognition of speech presented in the AV mode and to recognition of foreign-accented speech.

### Summary and Conclusion

This experiment examined integration of asynchronous AV native English and foreign-accented sentences by younger and older listeners, as manifested on detection and recognition tasks. Compared to younger listeners, older listeners are less sensitive to auditory lead asynchronies and perceive wider ranges of AV asynchronous sentences as synchronous, especially for NE talkers. These findings reflect possible age-related slowing of auditory speech streams in auditory lead/visual lag conditions and reduced sensitivity to asynchrony in auditory lag/visual lead conditions. In contrast, younger and older listeners with normal hearing and older listeners with hearing loss showed comparable patterns of AV speech integration, indicating that listener age and hearing loss did not impact recognition of asynchronous AV sentences. Although the AV simultaneity window determined from detection judgments was wider for NE talkers than NS talkers by older listeners, there were no differences in recognition performance for NE and NS talkers across a broad range of AV asynchronies. Overall, these findings suggest that unlike younger listeners, older listeners’ speech recognition may be negatively impacted by asynchronous AV speech stimuli that they judged as synchronous.

## Data Availability Statement

The original contributions presented in the study are publicly available. The data can be found here: http://hdl.handle.net/1903/28269.

## Ethics Statement

The studies involving human participants were reviewed and approved by University of Maryland Institutional Review Board. The patients/participants provided their written informed consent to participate in this study.

## Author Contributions

SG-S designed the experiments, oversaw data collection, analyzed data, and wrote the manuscript. MS assisted with stimulus creation, implemented and performed the experiments, and conducted some of the data analyses. KO collected and analyzed some of the data. GY-K was involved in designing the experiments and manuscript preparation. All authors contributed to the article and approved the submitted version.

## Conflict of Interest

The authors declare that the research was conducted in the absence of any commercial or financial relationships that could be construed as a potential conflict of interest.

## Publisher’s Note

All claims expressed in this article are solely those of the authors and do not necessarily represent those of their affiliated organizations, or those of the publisher, the editors and the reviewers. Any product that may be evaluated in this article, or claim that may be made by its manufacturer, is not guaranteed or endorsed by the publisher.
