# Supplementary material for: Detection and Recognition of Asynchronous Auditory/Visual Speech: Effects of Age, Hearing Loss, and Talker Accent
Source: Front Psychol. 2022 Jan 28;12:772867. doi: 10.3389/fpsyg.2021.772867 (PMC8832148; doi:10.3389/fpsyg.2021.772867)
Supplement: Supplementary file 1 [file Table_1.DOCX]

Supplementary Material

Table. Results of initial general linear mixed effects model of listener group, talker native language, and AV asynchrony conditions on detection of AV asynchrony (non-significant interaction effects were removed), referenced to NE talker, Asynchrony 0 ms, and YNH listeners. [Talker NS = native Spanish talker; OHI = older hearing-impaired listeners, ONH = older normal-hearing listeners.]

**Coefficient SE *z p***

------------------------------------------------------------------------------------------------------------------

Intercept 2.67 0.33 8.03 <.001

Talker NS 0.22 0.41 0.52 >.05

Async -450 -6.90 0.65 -10.55 <.001

Async -400 -6.17 0.51 -12.17 <.001

Async -350 -6.00 0.48 -12.43 <.001

Async -300 -6.00 0.48 -12.37 <.001

Async -250 -5.30 0.41 -12.84 <.01

Async -200 -4.39 0.36 -12.14 <.001

Async -150 -3.41 0.34 -10.06 <.001

Async -100 -2.03 0.33 -6.08 <.001

Async - 50 -1.00 0.36 -2.81 <.01

Async + 50 0.20 0.43 0.47 >.05

Async +100 -0.45 0.38 -1.19 >.05

Async +150 -1.14 0.35 -3.24 <.01

Async +200 -1.53 0.34 -4.49 <.001

Async +250 -2.00 0.33 -5.99 <.001

Async +300 -3.03 0.33 -9.05 <.001

Async +350 -4.06 0.35 -11.53 <.001

Async +400 -4.66 0.37 -12.48 <.001

Async +450 -5.30 0.41 -12.87 <.001

Grp OHI -0.36 0.44 -0.82 > .05

Grp ONH 0.68 0.56 1.22 >.05

Talker NS x Async -450 -11.78 3.55 -3.32 <.001

Async -300 x Grp OHI 1.48 0.60 2.47 <.05

Async -250 x GrpOHI 1.08 0.53 2.04 <.05

Async -200 x GrpOHI 1.03 0.47 2.18 <.05

Async +300 x GrpOHI 0.96 0.45 2.14 <.05

Async +350 x GrpOHI 1.00 0.46 2.17 <.05

Async +450 x GrpOHI 1.29 0.52 2.46 <.05

Talker NS x Asynch -450 x GrpOHI 9.70 3.60 2.69 <.01

Talker NS x Asynch +50 x GrpOHI -1.70 0.83 -2.05 <.05

Talker NS x Asynch-450 x Grp ONH 11.64 3.61 3.22 <.01

----------------------------------------------------------------------------------------------------------------
